# Pan-Cancer Prognostic Analysis of NMDAR Genes Discovered Therapeutic Implications of Neuronal–Cancer Crosstalk Mediator GRIN2A for Small Cell Lung Cancer

**DOI:** 10.3390/biomedicines14061196

**Published:** 2026-05-25

**Authors:** Jiaxun Zhang, Akezhouli Shahatiaili, Yuhan Hou, Ning Zhou, Ke Huang, Xiaojun Wang, Dongmei Wang, Zhentao Yu, Xiaoli Feng, Yibo Gao

**Affiliations:** 1 Department of Thoracic Surgery, National Cancer Center/National Clinical Research Center for Cancer/Cancer Hospital, Chinese Academy of Medical Sciences and Peking Union Medical College, Beijing 100021, China; zhangjiaxun2023@outlook.com (J.Z.); b2023003037@pumc.edu.cn (A.S.); 19916938790@163.com (K.H.); 2 Department of Pathology, National Cancer Center/National Clinical Research Center for Cancer/Cancer Hospital, Chinese Academy of Medical Sciences and Peking Union Medical College, Beijing 100021, China; 3 Department of Neurology, Nanfang Hospital, Southern Medical University, Guangzhou 510515, China; 4 Department of Thoracic Surgery, National Cancer Center/National Clinical Research Center for Cancer/Cancer Hospital & Shenzhen Hospital, Chinese Academy of Medical Sciences and Peking Union Medical College, Shenzhen 518116, China; 5 Central Laboratory & Shenzhen Key Laboratory of Epigenetics and Precision Medicine for Cancers, National Cancer Center/National Clinical Research Center for Cancer/Cancer Hospital & Shenzhen Hospital, Chinese Academy of Medical Sciences and Peking Union Medical College, Shenzhen 518116, China; 6 Laboratory of Translational Medicine, National Cancer Center/National Clinical Research Center for Cancer/Cancer Hospital, Chinese Academy of Medical Sciences and Peking Union Medical College, Beijing 100021, China; 7 State Key Laboratory of Molecular Oncology, National Cancer Center/National Clinical Research Center for Cancer/Cancer Hospital, Chinese Academy of Medical Sciences and Peking Union Medical College, Beijing 100021, China; 8 Department of Gastroenterology, Shanxi Province Cancer Hospital/Shanxi Hospital Affiliated to Cancer Hospital, Chinese Academy of Medical Sciences/Cancer Hospital Affiliated to Shanxi Medical University, Taiyuan 030013, China

**Keywords:** small cell lung cancer, NMDA receptor, GRIN2A, cancer neuroscience, tumor microenvironment, drug resistance

## Abstract

**Background:** As the most lethal neuroendocrine tumor, small cell lung cancer (SCLC) can drive its progression by hijacking neuronal mechanisms. At the core of this neural integration is the N-methyl-D-aspartate receptor (NMDAR) complex. However, its pan-cancer expression and clinical significance in SCLC remain poorly understood. **Methods:** We characterized NMDAR transcriptomic profiles across human cancers to develop the NMDAscore, and analyzed three independent European and Asian SCLC cohorts to identify prognostic biomarkers. Furthermore, we investigated the molecular mechanisms of GRIN2A and evaluated the efficacy of GluN2 inhibitors. **Results:** The developed NMDAscore exhibited significant prognostic correlations in ACC, COAD, KIRC, UVM, KIRP, OV, PCPG, UCS, THCA, THYM, HNSC, KICH, LGG, and PAAD. Focusing on the SCLC cohorts, we identified *GRIN2A* (encoding the GluN2A subunit) as a statistically relevant prognostic biomarker associated with poor survival. Mechanistically, *GRIN2A* upregulation correlates with the activation of neuro-synaptic signaling, metabolic reprogramming, genomic instability, and an immune-cold microenvironment characterized by CD8^+^ T cell exclusion. Pharmacological inhibition of GluN2 using dizocilpine and the FDA-approved antagonist memantine suppressed SCLC proliferation and tumorigenicity in vitro, in 3D tumor spheroids and in vivo xenograft models. **Conclusions:** Collectively, these findings establish *GRIN2A* as a prognostic biomarker, linking synaptic hijacking, metabolic plasticity, immune evasion, and drug resistance, and identify the therapeutic potentials of the GluN2 inhibitors dizocilpine and memantine for SCLC.

## 1. Introduction

The intricate bidirectional communication between tumors and the host nervous system has recently emerged as a critical hallmark of cancer. This paradigm shift, termed “cancer neuroscience,” reveals that solid tumors can actively hijack neural mechanisms to fuel their initiation, progression, and metastatic dissemination [1,2]. Central to this malignant neuromimicry is the exploitation of neurotransmitter signaling, particularly glutamatergic pathways, which conventionally govern synaptic plasticity and neuronal survival [3]. The N-methyl-D-aspartate receptor (NMDAR) is a principal ionotropic glutamate receptor complex that regulates calcium influx and downstream signaling cascades [4,5]. While aberrant NMDAR activity has been implicated in various neuropathologies, and foundational studies have reported ectopic NMDAR expression in certain human solid tumors [6], its systematic expression landscape, inter-tumoral heterogeneity, and broad prognostic implications across multiple malignancies remain poorly characterized.

To bridge this gap, our study first conducted pan-cancer analysis of NMDA receptor genes to systematically decode the collective expression profiles. We uncovered profound inter-tumoral heterogeneity, wherein NMDAR expression demonstrated highly context-dependent prognostic values across different human cancers. This pan-cancer landscape compellingly suggested that specific NMDAR components might serve as prognostic marker in tumors with inherent neural or neuroendocrine traits.

Consequently, we directed our focus toward Small Cell Lung Cancer (SCLC), a recalcitrant, high-grade neuroendocrine malignancy characterized by an exceptionally rapid doubling time, early dissemination, and a dismal five-year survival rate of less than 7% [7,8]. Our previous studies have demonstrated that the prognosis of SCLC is closely associated with ferroptosis-related genes, and that Hexokinase 2 (HK2) promotes cancer cell stemness in SCLC [9,10]. Groundbreaking studies published recently have demonstrated that SCLC cells are not electrically inert but possess intrinsic excitability, forming bona fide functional synapses with host neurons to hijack glutamatergic signaling [11,12,13]. However, the precise NMDAR subunits that dictate clinical outcomes and orchestrate this neuro-synaptic integration in SCLC populations are completely unknown.

By comprehensively evaluating SCLC cohorts (181 samples from three datasets), we identified *GRIN2A* (encoding the GluN2A subunit) as the uniquely crucial prognostic biomarker within the NMDAR family. We integrated transcriptomic, genomic, and tumor microenvironment analyses to systematically evaluate its associations with functional signaling pathways, genomic instability, and immune evasion. Furthermore, we performed in vitro, 3D spheroids, and in vivo experiments for repurposing GluN2 antagonists (dizocilpine and memantine) as a novel therapeutic strategy, highlighting the translational potential of targeting the specific neuron-cancer interface to overcome SCLC recalcitrance.

## 2. Materials and Methods

### 2.1. Public Data Acquisition and Processing

We obtained transcriptome profiles and sample data from TCGA (https://portal.gdc.cancer.gov/) and the Cancer Cell Line Encyclopedia (CCLE) database. Additionally, UCSC Xena databases (https://xenabrowser.net/datapages/, accessed on 20 December 2025) also provided most of these datasets we used. We collected several publicly available information on transcriptomics, genomics, and clinical data in SCLC. First, we downloaded the transcriptomic expression, somatic mutation, and clinical data of SCLC from George et al. [14] (*n* = 81, RNA-seq) and Qian Liu et al. [15] (*n* = 112, RNA-seq). The RNA-seq data (Illumina TruSeq) were transformed by log_2_(TPM + 1). Second, we extracted the expression profile and clinical data of SCLC in GSE60052 [16] (*n* = 79, RNA-seq) and GSE30219 [17] (*n* = 21, Affymetrix) from Gene Expression Omnibus (GEO, https://www.ncbi.nlm.nih.gov/geo/, accessed on 26 December 2025). Batch effects from non-biological technical biases were corrected using the “ComBat” algorithm of the “sva” package [18]. Data normalization and processing were performed using R version 4.3.1 software. Ethics approval and informed consent were not required.

### 2.2. Quantification of NMDAscore

NMDAscore was calculated as a gene-signature score based on *GRIN1*, *GRIN2A*, *GRIN2B*, *GRIN2C*, *GRIN2D*, *GRIN3A*, and *GRIN3B*. Briefly, TPM values were log2-transformed [log_2_(TPM + 1)], gene expression was z-scored for each gene across all samples, and the NMDAscore for each sample was defined as the mean z-score of these seven genes.

### 2.3. Differentially Expressed Gene Analysis and Prognostic Analysis

Differential expression analyses were performed using the limma R package with empirical Bayes moderation. For subsequent general differential gene expression analyses, statistical significance was set at an adjusted *p* < 0.05. To evaluate prognostic relevance, the optimal cutoff value for NMDAR genes mRNA expression was determined using the “surv_cutpoint” function, stratifying patients into high and low expression subgroups. Univariate Cox proportional hazards regression was conducted utilizing the survival package (v3.7.0) to calculate hazard ratios across four survival endpoints: OS, DSS, DFI, and PFI. Finally, Kaplan–Meier survival curves were generated using the “survfit” function, and statistical differences between the survival distributions were assessed via the log-rank test.

### 2.4. Gene Set Enrichment Analysis (GSEA)

To investigate the biological pathways associated with *GRIN2A* expression, GSEA was performed using the “fgsea” R package [19]. We utilized gene sets from the MSigDB database (https://www.gseamsigdb.org/gsea/msigdb/, accessed on 3 January 2026), including Hallmark, GO, and KEGG. Results were considered significant with a Normalized Enrichment Score (NES) and an adjusted *p*-value < 0.05.

### 2.5. Tumor Immune Microenvironment Analysis and Evaluation of Immune Checkpoint

The ESTIMATE algorithm [20] was applied to the bulk RNA-sequencing data to calculate the ImmuneScore, StromalScore, ESTIMATEScore, and TumorPurity for each SCLC sample. Next, the infiltration abundances of specific immune and stromal cell populations were estimated using three independent algorithms: xCell, TIMER, and EPIC. Additionally, the mRNA expression levels of selected immune checkpoint-related genes were extracted. All the aforementioned immune scores, cell infiltration proportions, and immune checkpoint expression levels were compared between the *GRIN2A*-high and *GRIN2A*-low expression groups using the Wilcoxon rank-sum test.

### 2.6. Drug Sensitivity Prediction

To evaluate the clinical implications of *GRIN2A* in chemotherapy and targeted therapy, we utilized the “oncoPredict” R package [21] to estimate the half-maximal inhibitory concentration (IC50) of drugs based on the GDSC database (https://www.cancerrxgene.org/) [22]. A total of 287 compounds were evaluated. The differences in drug sensitivity between the high- and low-*GRIN2A* expression groups were compared using the Wilcoxon rank-sum test. *p*-value < 0.05 was considered statistically significant.

### 2.7. Cell Lines and Cell Culture

The human SCLC cell lines NCI-H446 and NCI-H1048 were purchased from American Type Culture Collection (ATCC). NCI-H446 cells were cultured in RPMI 1640 medium (Corning, Corning, NY, USA) supplemented with 10% fetal bovine serum (FBS; Corning, NY, USA), 1% penicillin and streptomycin (Thermo Fisher Scientific, Waltham, MA, USA) in a humidified incubator at 37 °C with 5% CO_2_. NCI-H1048 cells were cultured in HITES complete medium (ZQXZBIO, Shanghai, China) supplemented with 5% FBS (Corning) in a humid incubator at 37 °C with 5% CO_2_. 

### 2.8. Cell Viability Assay

Cells were seeded in 96-well plates (10,000 cells per well) and treated after 24 h with drugs for 48 h. After removing the medium with drugs, 10 μL of Cell Counting Kit-8 reagent (MCE, Princeton, NJ, USA) and 90 μL of fresh medium were added to each well. After 2 h of incubation, absorbance was measured at 450 nm, and cell viability was calculated using GraphPad Prism software 8.4.3.

### 2.9. Cell Proliferation and Colony Formation Assays

Real-time cell proliferation was monitored using the Incucyte live-cell analysis system (Essen BioScience, Ann Arbor, MI, USA). Cells were seeded at a density of 10,000 cells per well in 96-well plates and treated with drugs. The seeding density was appropriate for the characteristically small cell volume of SCLC cells and to maintain logarithmic growth. Phase confluency was quantified over 72 h and cell count was calculated using GraphPad Prism software.

For EdU assay, cells were seeded at a density of 50,000 cells per well in 24-well plates and treated after 2 h with drugs for 48 h. Following the protocol of the EdU kit (C0071S, Beyotime, Shanghai, China), the cells were added with Edu reagent for 2 h. The cells were washed twice with 1× PBS for 5 min and incubated with 4% polyformaldehyde for 30 min. After washing twice with PBS for 5 min, the samples were permeated with 0.3% TritonX-100 in PBS, and stained with a reaction solution.

For colony formation assays, 2000 cells were seeded in 6-well plates with fresh cell culture medium. Then plates with cells were treated with drugs for 12 days. The plates were stained with crystal violet after fixation with 4% polyformaldehyde.

### 2.10. 3D Cell Line-Derived Spheroid Assay

Cells were seeded at a density of 100,000 cells per well in 24-well plates added with Honeycomb Chips (Vivoid, Suzhou, China) and allowed to form spheroid overnight. Drug treatment was added for 72 h. Then plates with spheroids were stained with PI solution (Beyotime) and Calcein Blue AM (Thermo Fisher Scientific) for live/dead fluorescence staining assay.

### 2.11. Cell Migration Assay

For Transwell migration, 100,000 cells were seeded in 200 μL of medium containing without FBS in the top chamber (8.0 μM pore size, Corning). Additionally, 600 μL of 20% FBS-containing medium was placed into the bottom chamber as an attractant. After incubation for 24 h, any cells that did not invade the lower side of the chamber were removed from the top side. Invasive cells located on the lower side of the chamber were fixed in 4% polyformaldehyde and stained with crystal violet.

### 2.12. RNA Extraction, cDNA Synthesis and Quantitative Real Time PCR (qRT–PCR)

To verify the success of GRIN2A knockdown assay at mRNA level, we performed qRT-PCR analysis. cDNA was prepared from 2 µg of RNA extracted from H1048 and H446 cells using TRIzol reagent (#15596026CN, Invitrogen, Carlsbad, CA, USA) and *EasyScript*^®^ One-Step gDNA Removal and cDNA Synthesis SuperMix (#AE311, TransGen, Beijing, China). RT-qPCR was conducted with the TransScript^®^ Green One-Step qRT-PCR SuperMix (#AQ211, TransGen, China) and an Eppendorf quantitative PCR instrument (Eppendorf, Hamburg, Germany). GRIN2A expression levels were normalized to GAPDH. Primer sequences for qRT-PCR are listed in Supplementary Table S1.

### 2.13. Cell Transfection

Following the manufacturer’s protocol, H048 and H446 cells seeded in 6-well plates, transfection was performed with 50 nM siRNA oligonucleotides when the cell confluence reached about 60%, siRNA synthesized by GenePharma Biotech (Shanghai, China). The GRIN2A siRNA sequences are provided in Supplementary Table S1. Lipofectamine™ 3000 transfection reagent (#L3000015, Thermo Fisher, Waltham, MA, USA) was used for the transfection.

### 2.14. Western Blotting

Proteins were extracted with RIPA lysis buffer (Yeasen, Shanghai, China), and total protein concentrations were quantified with the BCA Protein Quantification Kit (Yeasen, China) according to the manufacturer’s instructions. Proteins were then separated by SDS-PAGE electrophoresis and transferred to polyvinylidene fluoride (PVDF) membranes (Millipore, Burlington, MA, USA), and blocked with 5% skim milk (freshly prepared) for 1.5 h. The membranes were probed overnight with primary antibodies against human GRIN2A (#83465-2-RR, 1:1000, Proteintech, Rosemont, IL, USA), Cleaved-Caspase3 (#F2523, 1:1000, Selleck, Houston, TX, USA), cleaved-PARP (Asp214) (#F0136, 1:1000, Selleck), Phospho-p44/42 MAPK (Erk1/2) (T202/Y204) Antibody (#F0007, 1:1000, Selleck), Phospho-Akt (Ser473) (#4060, 1:1000, CST, Danvers, MA, USA), and β-Actin (#F0012, 1:10,000, Selleck). Afterward, membranes were subsequently incubated with horseradish peroxidase-conjugated secondary antibodies for 1 h at ordinary temperature. Protein bands were visualized using an ECL Chemiluminescence Detection Kit (Beyotime, Shanghai, China) and captured with an imaging system.

### 2.15. Mouse Model

Female 4- to 6-week-old BALB/c nude mice were used for this study. Animal care and treatment followed institutional guidelines. A total of 5 × 10^6^ H1048 cells in 100 µL of PBS were subcutaneously injected into the right flank of six-week-old female BALB/c nude mice. Seven days post-implantation, mice were randomized into three groups (*n* = 5 per group) and treated with 0.9% normal saline as a control, dizocilpine (0.2 mg/kg, i.p. daily), or memantine (25 mg/kg, p.o. daily). Tumor volume was measured every two days using calipers and calculated as: tumor volume = (length × width^2^)/2. After 25 days, mice were euthanized in accordance with institutional guidelines.

### 2.16. Statistical Analysis

All statistical analyses were conducted using R version 4.3.1 and GraphPad Prism 8.4.3. Hazard ratios were computed with a univariate Cox regression model. Pearson correlation analysis was applied to evaluate relationships among variables with non-normal distributions. We adjusted *p*-values, including the false discovery rate, using *t*-test, two-way ANOVA, and the Wilcoxon method. At *p* < 0.05, statistical significance was recognized. Significance ranking is indicated throughout this report as follows: **** *p* < 0.0001, *** *p* < 0.001, ** *p* < 0.01, * *p* < 0.05, and non-significant (ns).

## 3. Results

### 3.1. Pan-Cancer Expression Landscape of NMDA Receptor Genes

**Figure 1:**
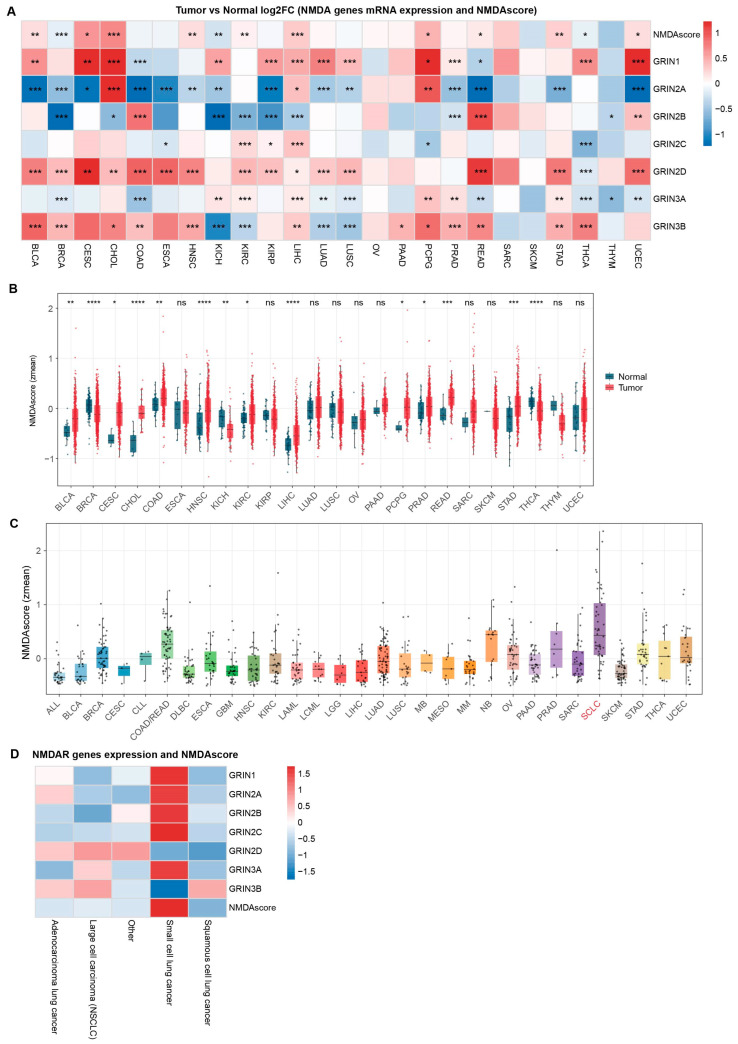
Integrative pan-cancer analysis of expression landscape of NMDA receptor genes. (**A**) Heatmap illustrating the differential expression of NMDA receptor genes and the calculated NMDAscore between tumor tissues and corresponding adjacent normal tissues across the TCGA cohorts. The color gradient represents the log_2_(Fold Change) values. (**B**) Boxplot illustrating the differential mRNA expression of NMDAscore between tumor tissues and corresponding normal tissues across the TCGA pan-cancer cohorts. (**C**) Comparison of the NMDAscore across various cancer cell lines based on the CCLE database. The red color represents the cancer types with the highest NMDAscores. (**D**) Heatmap illustrating the expression patterns of NMDAR genes and the NMDAscore across different histological subtypes of lung tumors. The “Large cell carcinoma (NSCLC)” group refers to CCLE/DepMap-annotated cell lines classified as “Lung: NSCLC_Large_Cell” or “large_cell_carcinoma” and does not specifically represent large-cell neuroendocrine carcinoma (LCNEC). The “Other” group includes lung cancer cell lines not clearly classified as adenocarcinoma, squamous cell carcinoma, SCLC, or large cell carcinoma according to the original CCLE/DepMap annotation. * *p* < 0.05, ** *p* < 0.01, *** *p* < 0.001, **** *p* < 0.0001, ns = not significant.

To systematically investigate the expression patterns of N-methyl-D-aspartate (NMDA) receptor genes across multiple malignancies, we integrated transcriptomic data from The Cancer Genome Atlas (TCGA) databases. To quantify the collective expression profile of these genes, an “NMDAscore” was computed for each sample. Our pan-cancer analysis revealed widespread differential expression of the NMDAscore between tumor and corresponding adjacent normal tissues. Specifically, the NMDAscore was significantly elevated in bladder urothelial carcinoma (BLCA), cervical squamous cell carcinoma and endocervical adenocarcinoma (CESC), cholangiocarcinoma (CHOL), head and neck squamous cell carcinoma (HNSC), kidney renal clear cell carcinoma (KIRC), liver hepatocellular carcinoma (LIHC), pheochromocytoma and paraganglioma (PCPG), rectum adenocarcinoma (READ), stomach adenocarcinoma (STAD), and uterine corpus endometrial carcinoma (UCEC). Conversely, a marked downregulation of the NMDAscore was observed in breast invasive carcinoma (BRCA), kidney chromophobe (KICH), and thyroid carcinoma (THCA) (Figure 1A,B).

To further investigate the intrinsic baseline of NMDA receptor activity across different malignancies, we evaluated the NMDAscore in various cancer cell lines using the Cancer Cell Line Encyclopedia (CCLE) database. SCLC cell lines exhibited exceptionally high NMDAscores, ranking among the highest across all analyzed malignancies. This suggests a uniquely active NMDA pathway intrinsic to SCLC cells (Figure 1C). To determine the specificity of this signature within the context of pulmonary malignancies, we examined the expression profiles of NMDA receptor genes and NMDAscore across different histological subtypes of lung tumors. The heatmap analysis revealed a profound and specific upregulation of both individual NMDAR genes (including *GRIN1*, *GRIN2A-C*, and *GRIN3A*) and the aggregate NMDAscore in SCLC (Figure 1D). In distinct contrast, other lung cancer subtypes, such as adenocarcinoma, squamous cell carcinoma, and large cell lung cancer, displayed broadly negative or low enrichment. Taken together, these findings robustly demonstrate that the NMDA receptor system is highly enriched in SCLC, highlighting its potential specific biological importance in this aggressive lung cancer subtype.

### 3.2. Clinical Correlation Analysis of NMDA Receptor Genes

**Figure 2:**
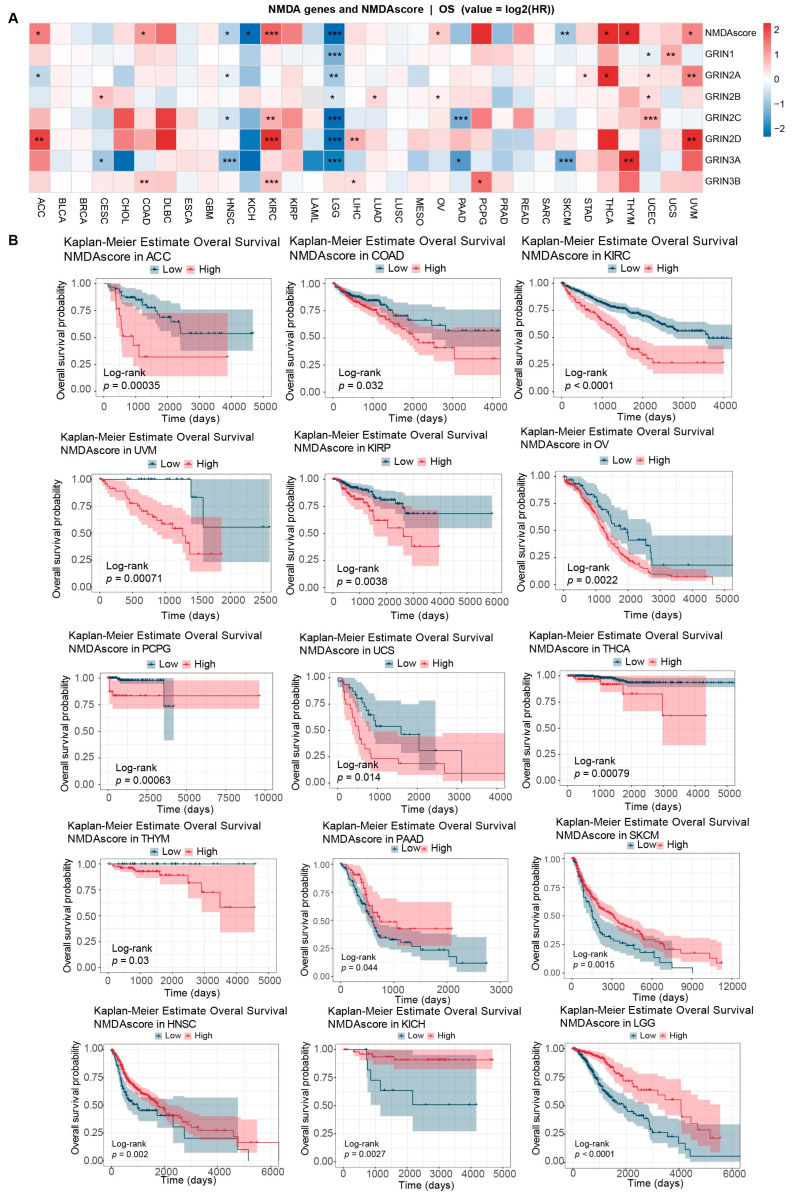
Pan-cancer overall survival prognostic value of NMDA receptor genes. (**A**) Heatmap depicting the univariate Cox regression analysis of the NMDAscore for Overall Survival (OS) across multiple malignancies. The color scale indicates the log_2_(Hazard Ratio). (**B**) Kaplan–Meier survival curves of OS for patients stratified by high and low NMDAscores in fifteen representative cancer cohorts (ACC, COAD, KIRC, UVM, KIRP, OV, PCPG, UCS, THCA, THYM, PAAD, SKCM, HNSC, KICH, and LGG). The log-rank test was used to calculate *p*-values. * *p* < 0.05, ** *p* < 0.01, *** *p* < 0.001.

To evaluate the clinical relevance of NMDA receptor genes, we performed univariate Cox regression analyses to determine the association between the NMDAscore and various survival endpoints. This comprehensive assessment revealed that the NMDAscore serves as a robust prognostic indicator across diverse cancer types. For Disease-Free Interval (DFI), a higher NMDAscore correlated significantly with poor clinical outcomes, presenting elevated hazard ratios (HRs) in prostate adenocarcinoma (PRAD) and STAD (Supplementary Figure S1A). Similarly, an increased NMDAscore was identified as a significant risk factor for Progression-Free Interval (PFI) in adrenocortical carcinoma (ACC), colon adenocarcinoma (COAD), KIRC, PCPG, PRAD, and uveal melanoma (UVM) (Supplementary Figure S1B). Regarding Disease-Specific Survival (DSS), the NMDAscore demonstrated strong prognostic value in ACC, COAD, KIRC, kidney renal papillary cell carcinoma (KIRP), PCPG, STAD, THCA, and UVM (Supplementary Figure S1C). Furthermore, elevated NMDAscores were significantly associated with shorter Overall Survival (OS) in ACC, COAD, KIRC, ovarian serous cystadenocarcinoma (OV), THCA, thymoma (THYM), and UVM (Figure 2A).

To further validate these prognostic associations, Kaplan–Meier (KM) survival analyses were conducted using TCGA clinical cohorts. Consistent with the univariate Cox regression results, the KM curves illustrated that patients stratified into the high-NMDAscore group exhibited significantly poorer OS in ACC, COAD, KIRC, UVM, KIRP, OV, PCPG, uterine carcinosarcoma (UCS), THCA, and THYM (Figure 2B). In contrast, an elevated NMDAscore predicted a more favorable OS in HNSC, KICH, brain lower grade glioma (LGG), pancreatic adenocarcinoma (PAAD), and skin cutaneous melanoma (SKCM) (Figure 2B). Taken together, these data underscore the pronounced inter-tumoral heterogeneity of NMDA receptor expression and highlight its context-dependent prognostic implications across human cancers.

### 3.3. Identification of GRIN2A as a Crucial Prognostic Biomarker in SCLC

**Figure 3:**
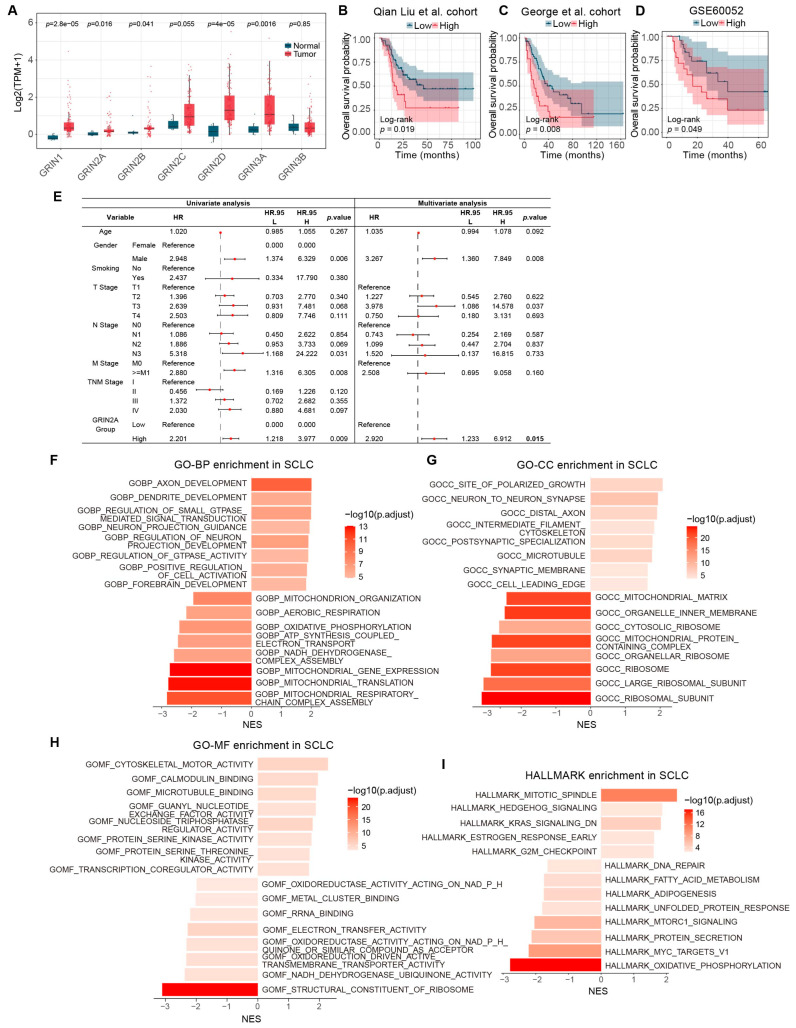
Expression profile, clinical significance, and functional enrichment analysis of *GRIN2A* in SCLC. (**A**) Boxplots displaying the mRNA expression levels of NMDAR family genes (*GRIN1*, *GRIN2A-D*, *GRIN3A-B*) in SCLC tumor tissues compared to adjacent normal tissues. (**B**) Kaplan–Meier survival curve of OS for SCLC patients stratified by high and low *GRIN2A* expression in the Qian Liu et al. SCLC cohort. (**C**) Kaplan–Meier survival curve of OS for SCLC patients stratified by high and low *GRIN2A* expression in George et al. SCLC cohort. (**D**) Kaplan–Meier survival curve of OS for SCLC patients stratified by high and low *GRIN2A* expression in the GSE60052 SCLC cohort. (**E**) Univariate and multivariate Cox regression analysis of *GRIN2A*. (**F**–**I**) Bar plots displaying the results of Gene Set Enrichment Analysis (GSEA) comparing high and low *GRIN2A* expression groups. The Normalized Enrichment Score (NES) and −log_10_(p.adjust) values are shown for (**F**) GO-BP, (**G**) GO-CC, (**H**) GO-MF, and (**I**) HALLMARK pathways. Statistical significance was determined using the log-rank test.

Given the pronounced neuroendocrine features characteristic of SCLC, we hypothesized that NMDAR genes might play a critical role in its progression. Therefore, we specifically evaluated the expression profiles of NMDAR family genes in SCLC cohorts. Our analysis revealed that the mRNA expression levels of *GRIN1*, *GRIN2A*, *GRIN2B*, and *GRIN2D* were significantly upregulated in SCLC tumor tissues compared to adjacent non-tumor tissues (Figure 3A). To further screen for genes with clinical relevance, we performed KM survival analysis. Strikingly, among the NMDAR family members, only high expression of *GRIN2A* was significantly associated with worse OS in SCLC patients both in the Qian Liu et al. cohort, the George et al. cohort and the GSE60052 cohort, whereas the other NMDAR genes showed no significant prognostic correlation (Figure 3B–D and Supplementary Figure S2). Univariate and multivariate Cox regression analysis further confirm that *GRIN2A* is an independent prognostic biomarker of SCLC (HR = 2.920, 95%CI = 1.233–6.912, *p* = 0.015) (Figure 3E).

To provide a broader context for the role of *GRIN2A*, we expanded our analysis to a pan-cancer level using TCGA datasets. Consistent with its dysregulation in SCLC, *GRIN2A* exhibited aberrant expression across various malignancies. Specifically, *GRIN2A* was significantly upregulated in CHOL, LIHC, and PCPG, while being downregulated in BLCA, BRCA, CESC, COAD, ESCA, HNSC, KICH, KIRP, LUAD, LUSC, PRAD, READ, STAD, and UCEC compared to normal tissues (Supplementary Figure S3A). In terms of pan-cancer prognosis, high *GRIN2A* expression predicted worse OS in multiple cancers, including BLCA, KIRP, LUSC, COAD, STAD, UCEC, THCA, and UVM (Supplementary Figure S3B). Conversely, elevated *GRIN2A* was associated with a more favorable OS in CHOL, KICH, ACC, MESO, HNSC and BRCA (Supplementary Figure S3C). Based on these collective findings, *GRIN2A* was selected as the core target for our subsequent functional and mechanistic investigations in SCLC.

### 3.4. Functional Enrichment Analysis Reveals GRIN2A-Associated Signaling Pathways

To elucidate the underlying biological functions and molecular mechanisms driving the prognostic difference in *GRIN2A* in SCLC, we stratified patients into high- and low-expression groups based on the median *GRIN2A* mRNA levels and performed Gene Set Enrichment Analysis (GSEA). KEGG pathway analysis demonstrated that the high-*GRIN2A* group was significantly enriched in the *EML4-ALK* fusion kinase pathway, PLCG-ERK signaling, and ADRB3-UCP1 signaling. In contrast, pathways related to translation initiation and mitochondrial electron transfer in complex I were markedly downregulated (Supplementary Figure S4).

Consistently, Gene Ontology (GO) analysis highlighted distinct functional profiles. For Biological Processes (BP), genes upregulated in the high-*GRIN2A* group were predominantly involved in neurogenesis and signal transduction, including axon development, dendrite development, neuron projection development, and small GTPase mediated signal transduction. Conversely, metabolic processes such as mitochondrial respiratory chain complex assembly, mitochondrial translation, aerobic respiration, and oxidative phosphorylation were significantly suppressed (Figure 3E). Similarly, Cellular Component (CC) analysis revealed that *GRIN2A* was closely associated with synaptic structures, such as neuron-to-neuron synapse, postsynaptic specialization, and synaptic membrane, whereas ribosomal subunits and mitochondrial matrix components were downregulated (Figure 3F). In terms of Molecular Function (MF), the high-expression group exhibited enrichment in microtubule binding, nucleoside triphosphatase regulator activity, and serine/threonine kinase activity, while activities related to structural constituents of ribosome, NADH dehydrogenase, electron transfer, and oxidoreductase acting on NADPH were downregulated (Figure 3G).

Furthermore, HALLMARK pathway analysis corroborated these findings, indicating a proliferative and aggressive phenotype in *GRIN2A*-high tumors. Specifically, pathways associated with cell cycle progression and oncogenic signaling, including mitotic spindle, Hedgehog signaling, *KRAS* signaling, and G2M checkpoint, were significantly activated. On the other hand, metabolism-related pathways, including oxidative phosphorylation, protein secretion, mTORC1 signaling, fatty acid metabolism, and DNA repair, were suppressed (Figure 3H).

Comparative GSEA indicated that high *GRIN2A* expression was uniquely and strongly enriched in critical oncogenic cascades, including epithelial–mesenchymal transition, KRAS, TGF-β, Wnt/β-catenin, and angiogenesis signaling (Supplementary Figure S5C).

Collectively, these results suggest that *GRIN2A* may promote SCLC progression by maintaining neuroendocrine differentiation and activating proliferative signaling cascades (e.g., *ERK* and *KRAS*), while concurrently reprogramming mitochondrial metabolism.

### 3.5. Genomic Alteration Landscape Associated with GRIN2A Expression in SCLC

**Figure 4:**
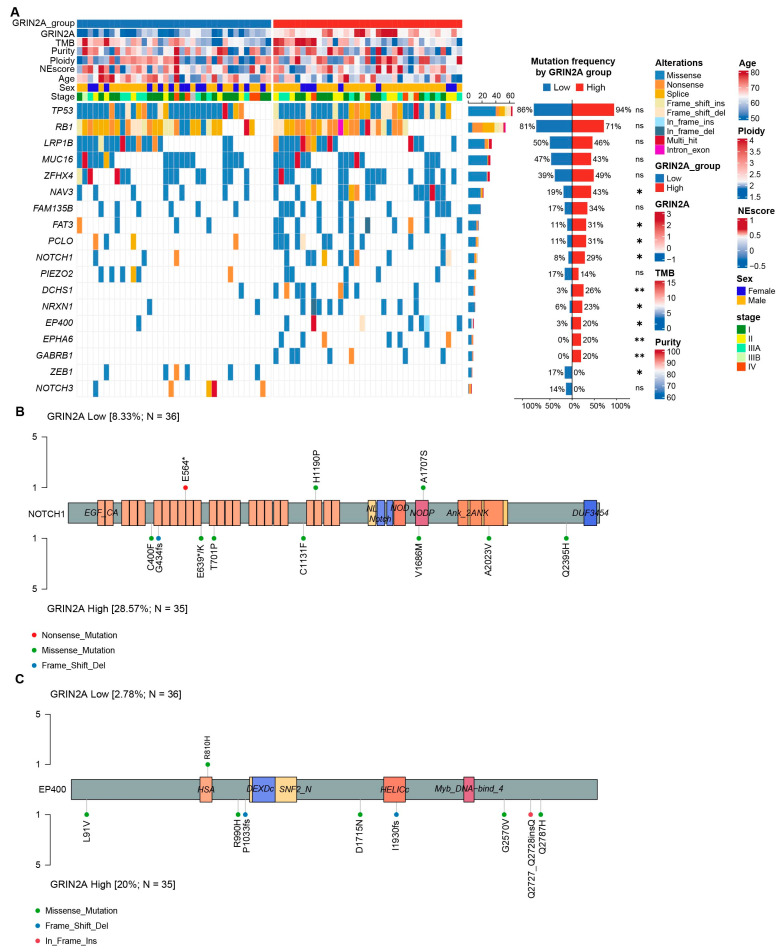
Genomic alteration landscape associated with *GRIN2A* expression in SCLC. (**A**) Oncoplot displaying the somatic mutation landscape and overall mutation frequencies of specific genes in the SCLC cohort. Different colors represent distinct variant classifications. Bar chart comparing the somatic mutation frequencies of frequently mutated genes between the *GRIN2A*-high and *GRIN2A*-low groups. (**B**,**C**) Lollipop plots mapping the distribution of somatic mutation sites and variant types for (**B**) *NOTCH1* and (**C**) *EP400* in patients stratified by high and low *GRIN2A* expression. Statistical significance of mutation frequencies was evaluated using Fisher’s exact test. * *p* < 0.05, ** *p* < 0.01.

We next characterized the somatic mutation landscape of NMDAR family genes to explore the genetic features of SCLC patients. Mutational landscape analyses demonstrated that *GRIN2A* harbored the highest frequency of recurrent somatic alterations among NMDAR genes in both the George (9.85%) and Qian Liu (8.04%) cohorts (Supplementary Figure S5B). To further elucidate the potential genetic mechanisms linked to *GRIN2A* dysregulation, we compared the somatic mutation profiles between the *GRIN2A*-high and *GRIN2A*-low expression groups. Notably, the *GRIN2A*-high group demonstrated a significantly higher mutation frequency of *NAV3*, *FAT3*, *PCLO*, *NOTCH1*, *DCHS1*, *NRXN1*, *EP400*, *EPHA6*, and *GABRB1*, compared to the low-expression cohort. Conversely, the mutation rates of *ZEB1* were markedly decreased in the high-*GRIN2A* group (Figure 4A). Detailed mapping of the mutation sites further illustrated these distinct genomic patterns, highlighting divergent mutational distributions for *NOTCH1* (Figure 4B) and *EP400* (Figure 4C) between the two subgroups. Collectively, these findings suggest that elevated *GRIN2A* expression is closely coupled with a specific genomic mutational profile, which may synergistically drive genomic instability and the malignant progression of SCLC.

### 3.6. GRIN2A Correlates with Suppressed Anti-Tumor Immunity and Poor Immunotherapy Response in SCLC

**Figure 5:**
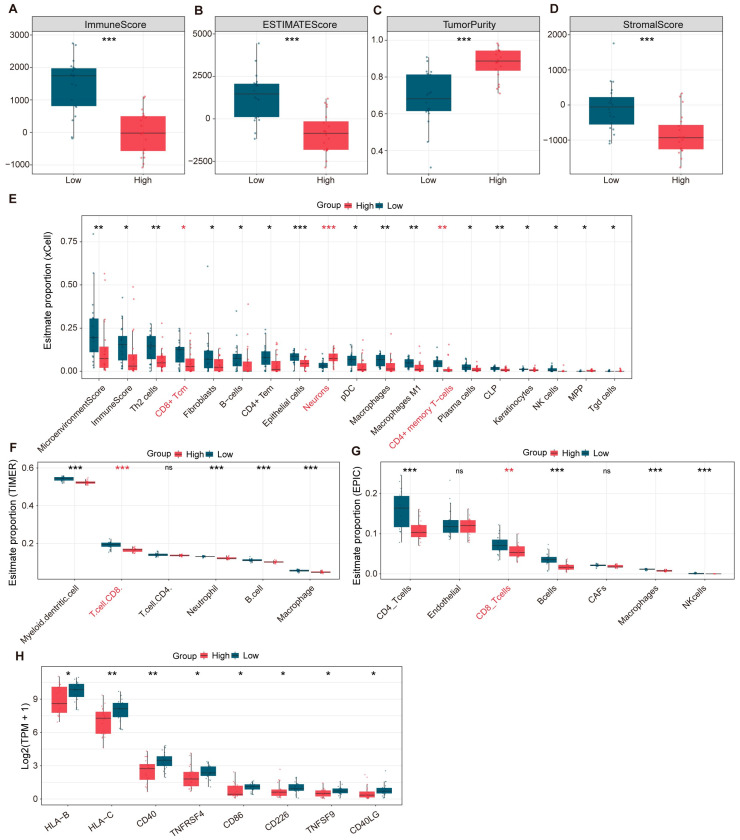
Immune landscape of high and low *GRIN2A* expression groups. (**A**–**D**) Boxplots comparing the (**A**) ImmuneScore, (**B**) ESTIMATEScore, (**C**) TumorPurity, and (**D**) StromalScore between the *GRIN2A*-high and *GRIN2A*-low expression groups. The scores were calculated utilizing the ESTIMATE algorithm. (**E**–**G**) Boxplots illustrating the relative abundance of infiltrating immune and stromal cell populations between the *GRIN2A*-high and *GRIN2A*-low groups, estimated by the (**E**) xCell, (**F**) TIMER, and (**G**) EPIC algorithms. Red color indicated the cell types associated with CD8+ T cells and neural features. (**H**) Boxplots comparing the mRNA expression levels of selected immune checkpoint-related genes (*HLA-B*, *HLA-C*, *CD40*, *TNFRSF4*, *CD86*, *CD226*, *TNFSF9*, and *CD40LG*) between the two subgroups. Statistical differences between the two groups were determined using the Wilcoxon rank-sum test. * *p* < 0.05, ** *p* < 0.01, *** *p* < 0.001.

Functional enrichment analysis suggested a potential role for *GRIN2A* in regulating the SCLC tumor immune microenvironment (TME). Therefore, we utilized the ESTIMATE algorithm to evaluate immune cell infiltration patterns in the *GRIN2A*-high and -low expression groups. The results showed that the *GRIN2A*-high group exhibited significantly lower Immune Scores, Estimate Scores, and Stromal Scores, whereas Tumor Purity was significantly higher compared to the low-expression group (Figure 5A–D). This suggests that high *GRIN2A* expression is associated with an overall “cold” immune microenvironment. Specifically, detailed composition analysis using the xCell algorithm revealed that the *GRIN2A*-high group was associated with a higher proportion of neurons, whereas the proportions of anti-tumor immune cells, such as CD8^+^ Tcms and CD4^+^ memory T cells, were significantly lower (Figure 5E). To validate this observation, we employed the TIMER and EPIC algorithm, which consistently confirmed a reduced proportion of CD8^+^ T cells in the *GRIN2A*-high group (Figure 5F,G). These findings suggest that high *GRIN2A* expression is indicative of a compromised anti-tumor immune response and potential CD8^+^ T cell exhaustion.

Furthermore, we examined the expression of immune checkpoints related to CD8^+^ T cell exhaustion. Compared to the low-expression group, the *GRIN2A*-high group exhibited decreased expression of HLA-B, HLA-C, CD40, TNFRSF4, CD86, CD226, TNFSF9, and CD40LG, indicating elevated CD8^+^ T cell exhaustion (Figure 5H). Collectively, these results indicate that *GRIN2A* emerges as a potential therapeutic target for SCLC, capable of decreasing CD8^+^ T cell infiltration and modulating anti-tumor immunity.

### 3.7. GRIN2A Expression Correlates with Sensitivity to Chemotherapeutic and Targeted Agents

**Figure 6:**
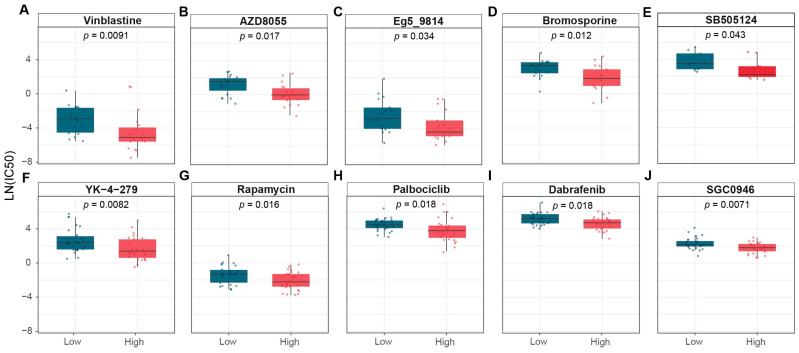
Evaluation of GRIN2A expression and IC50 for clinical medications. (**A**) Vinblastine, (**B**) AZD8055, (**C**) Eg5_9814, (**D**) Bromosporine, (**E**) SB505124, (**F**) YK-4-279, (**G**) Rapamycin, (**H**) Palbociclib, (**I**) Dabrafenib, (**J**) SGC0946.

Therapeutic resistance remains a major hurdle in clinical management and significantly contributes to poor prognosis in cancer patients. To explore the clinical implications of GRIN2A in SCLC treatment, we investigated the relationship between *GRIN2A* expression and anti-tumor drug sensitivity using the Genomics of Drug Sensitivity in Cancer (GDSC) database [22]. Our analysis using the oncoPredict algorithm [21] revealed distinct drug response patterns associated with GRIN2A levels. Cell lines with high GRIN2A expression exhibited significantly increased sensitivity to agents targeting mitosis (vinblastine), PI3K/MTOR signaling (AZD8055 and rapamycin), KSP11 (Eg5-9814), chromatin (bromosporine), RTK signaling (SB505124), RNA helicase A (YK-4-279), cell cycle (palbociclib), ERK/MAPK signaling (dabrafenib), and chromatin histone methylation (SGC0946) (Figure 6A–J). Taken together, these findings suggest that *GRIN2A* expression significantly influences sensitivity to various clinical agents, highlighting its potential as a predictive biomarker for guiding personalized treatment strategies and improving patient outcomes.

### 3.8. Pharmacological Inhibition of GluN2 Suppresses SCLC Proliferation and Migration In Vitro

**Figure 7:**
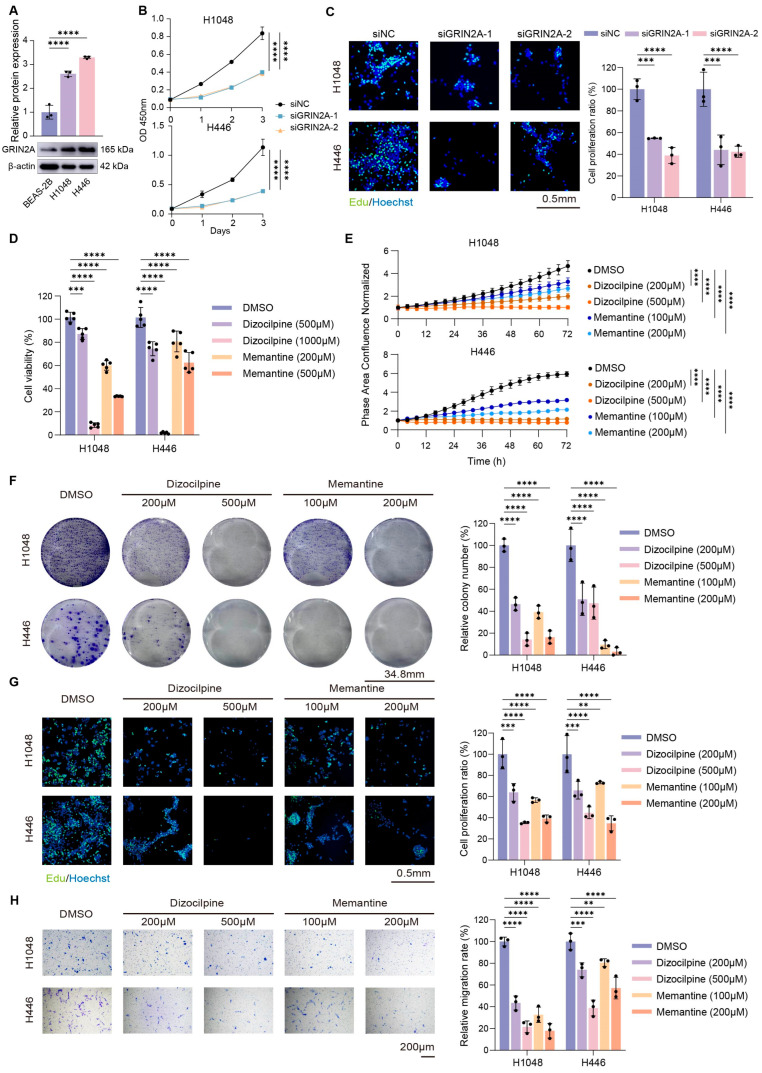
Targeting GluN2 suppress the proliferation and migration of SCLC cells in vitro. (**A**) Western blot analysis comparing the basal protein expression of GRIN2A between the normal human bronchial epithelial cell line BEAS-2B and SCLC cells (*n* = 3 per group). (**B**) CCK8 assay in H1048 and H446 cell lines after *GRIN2A* knockdown (*n* = 3 per group). (**C**) EdU assays in H1048 and H446 cell lines after *GRIN2A* knockdown (*n* = 3 per group). (**D**) Cell viability determined by CCK8 assay in H1048 and H446 cell lines treated with dizocilpine (500 μM and 1000 μM) and memantine (200 μM and 500 μM) for 48 h (*n* = 5 per group). (**E**) H1048 and H446 cell lines were treated with dizocilpine (500 μM and 1000 μM) and memantine (200 μM and 500 μM), and fold change in cell count was determined throughout 72 h. Two-way ANOVA was used to calculate the statistical significance for each time point and for each drug concentration (*n* = 6 per group). (**F**) Colony formation assays in H1048 and H446 cell lines treated with dizocilpine (200 μM and 500 μM) and memantine (100 μM and 200 μM) for 12 days. Relative colony numbers were shown on the right (*n* = 3 per group). (**G**) EdU assays in H1048 and H446 cell lines treated with dizocilpine (200 μM and 500 μM) and memantine (100 μM and 200 μM) for 48 h. Cell proliferation ratios were shown on the right (*n* = 3 per group). (**H**) Cell migration determined by Transwell assay in H1048 and H446 cell lines treated with dizocilpine (200 μM and 500 μM) and memantine (100 μM and 200 μM) (*n* = 3 per group). Statistical analysis includes two-tailed *t*-test; **** *p* < 0.0001, *** *p* < 0.001, and ** *p* < 0.01. Data presented as mean ± SD.

To further substantiate the tumor-specific role of *GRIN2A*, we evaluated its baseline protein expression across cell lines. Western blot analysis revealed that GRIN2A protein levels were markedly elevated in SCLC cells compared to the normal human bronchial epithelial cell line BEAS-2B (Figure 7A). To directly investigate the biological function of *GRIN2A* in SCLC, we performed *GRIN2A*-directed perturbation experiments using small interfering RNA (siRNA). The knockdown efficiency of *GRIN2A* in SCLC cells was verified by RT-qPCR (Supplementary Figure S6A) and Western blot analysis (Supplementary Figure S6B). Functionally, CCK8 assays demonstrated that silencing *GRIN2A* significantly inhibited SCLC cell proliferation (Figure 7B). Consistent with this, EdU incorporation assays revealed a marked decrease in DNA replication activity following GRIN2A knockdown (Figure 7C). Collectively, these perturbation results indicate that GRIN2A plays an oncogenic role in promoting SCLC progression.

To functionally validate the oncogenic role of GRIN2A in SCLC, we employed two GluN2 antagonists: dizocilpine and memantine (an FDA-approved drug for Alzheimer’s disease) [23,24]. We evaluated the therapeutic potential of targeting GluN2 by assessing cell proliferation and migration in SCLC cell lines (H1048 and H446) using CCK-8, IncuCyte, colony formation, EdU, and transwell assays.

To establish the rationale behind our drug concentration settings, we first evaluated the effects of lower doses of NMDAR antagonists. CCK-8 assays demonstrated that treatment with 50 µM or 100 µM of dizocilpine or memantine failed to effectively inhibit the viability of H1048 and H446 cells (Supplementary Figure S6C). CCK-8 and IncuCyte analyses revealed that treatment with dizocilpine or memantine significantly compromised cell viability in both H1048 and H446 cell lines (Figure 7A,B; *p* < 0.05). Consistent with these findings, pharmacological inhibition of GluN2 markedly suppressed long-term cell growth in colony formation assays (Figure 7C; *p* < 0.05) and impaired DNA replication activity as evidenced by reduced EdU incorporation (Figure 7D; *p* < 0.05). Furthermore, Transwell assays demonstrated that both antagonists significantly attenuated the migratory capacity of SCLC cells (Figure 7E; *p* < 0.05). Notably, the inhibitory effects of dizocilpine and memantine on SCLC proliferation and migration were observed to be dose-dependent (Figure 7A–E).

To evaluate potential off-target toxicity, we assessed the effects of dizocilpine and memantine on the normal human bronchial epithelial cell line BEAS-2B. CCK-8 assays demonstrated that the effective anti-tumor concentrations of these drugs induced no significant cytotoxicity in BEAS-2B cells (Supplementary Figure S6D), confirming a favorable therapeutic window.

### 3.9. Pharmacological Inhibition of GluN2 Impairs SCLC Tumorigenicity in 3D Spheroids and Xenograft Models

**Figure 8:**
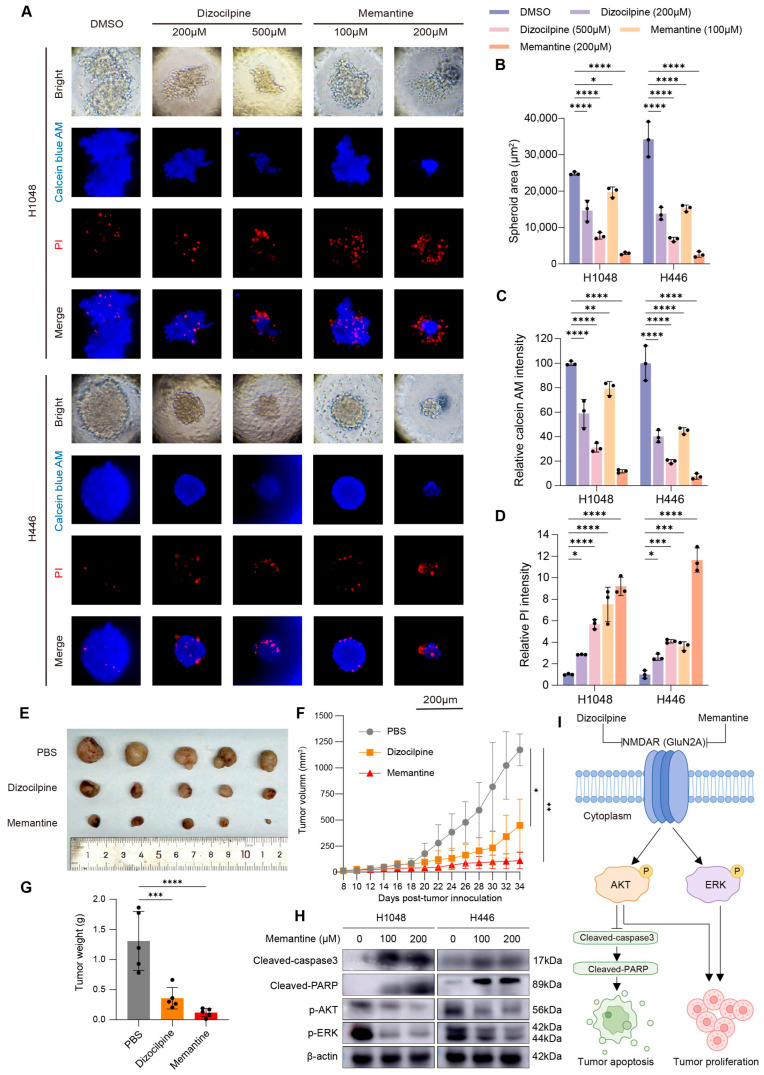
GluN2 antagonists suppress the progression of SCLC in 3D culture and in vivo. (**A**–**D**) H1048 (**A**) and H446 (**A**) cell line-derived spheroids were treated with dizocilpine (200 μM and 500 μM) and memantine (100 μM and 200 μM) for 72 h. Blue fluorescence (Calcein Blue AM) indicates lived cells, and red fluorescence (PI) indicates dead cells. Spheroid area (**B**), relative calcein blue AM intensity (**C**) and relative PI intensity (**D**) were shown on the right (*n* = 3 per group). (**E**–**G**) Subcutaneous tumors developed from H1048 cells treated with PBS, dizocilpine, and memantine respectively. Tumor volumes (**F**) and tumor weights (**G**) were measured. (**H**) Western blot analysis of p-ERK, p-AKT, cleaved-Caspase 3, and cleaved-PARP in H1048 and H446 cell lines treated with memantine. (**I**) A schematic model illustrating the mechanism by which targeting the GRIN2A subunit suppresses SCLC progression. Arrows indicate activation, whereas flat-headed arrows indicate inhibition. Statistical analysis includes two-tailed *t*-test; **** *p* < 0.0001, *** *p* < 0.001, ** *p* < 0.01, and * *p* < 0.05. Data presented as mean ± SD.

To evaluate the efficacy of GluN2 antagonists in a more physiologically relevant setting, we assessed the sphere-forming ability of H1048 and H446 cells using 3D tumor spheroid models, which better mimic in vivo cell–cell interactions [25] (Figure 8A). Quantitative analysis revealed that treatment with dizocilpine or memantine significantly suppressed spheroid formation in a dose-dependent manner, as evidenced by the marked reduction in spheroid area (Figure 8B; *p* < 0.05). Furthermore, we performed live/dead fluorescence staining to assess cell viability within the spheroids, using Calcein Blue AM to label viable cells (blue) and Propidium Iodide (PI) to label dead cells (red) (Figure 8A). Consistent with the morphological changes, we observed a dose-dependent decrease in the proportion of viable cells and a concurrent increase in cell death following treatment with either antagonist (Figure 8C,D; *p* < 0.05).

To validate the therapeutic potential of targeting GluN2 in vivo, we established a cell-derived xenograft (CDX) model by subcutaneously injecting H1048 cells into nude mice. As shown in Figure 8E–G, administration of dizocilpine or memantine significantly inhibited tumor growth, resulting in markedly reduced tumor volume and weight compared to the PBS control group. Collectively, these data provide compelling evidence that GRIN2A drives SCLC progression and that the pharmacological targeting of this receptor exerts potent anti-tumor effects both in vitro and in vivo.

To elucidate the downstream molecular mechanisms underlying the anti-tumor efficacy of memantine, we evaluated alterations in critical signaling cascades via Western blot. Memantine treatment significantly downregulated both the ERK and AKT signaling pathways in SCLC cells (Figure 8H). Concomitantly, the treatment induced robust cellular apoptosis, evidenced by the markedly increased accumulation of cleaved-Caspase 3 and cleaved-PARP. Finally, we summarized the proposed pharmacological mechanisms in a schematic model (Figure 8I), illustrating that targeting the GRIN2A subunit with memantine dismantles essential survival cascades and active apoptosis to suppress SCLC progression.

## 4. Discussion

NMDAR is a principal ionotropic glutamate receptor complex that regulates calcium influx and neurotransmitter signaling cascades, which is closely associated with anti-NMDAR encephalitis. Our previous studies have mapped the proteomic landscape of this disease, explored its prognostic markers, indicated its correlations with paroxysmal sympathetic hyperactivity, and expanded novel surgical operations [26,27,28]. The stagnation in survival outcomes for SCLC over the past three decades highlights a critical need to transcend traditional cytotoxic strategies and identify precise molecular dependencies [8]. Recent landmark studies revealed that SCLC cells exhibit “neuromimicry” by forming synapses to hijack glutamatergic signaling [11,12]. In this study, we systematically interrogated the role of the NMDAR complex starting from a macroscopic pan-cancer perspective. By formulating the NMDAscore, we demonstrated that NMDAR genes exhibit widespread but highly context-dependent dysregulation across multiple malignancies. Building upon this pan-cancer blueprint, we zoomed in on SCLC—a canonical neuroendocrine tumor—and identified *GRIN2A* as a master prognostic marker. Our findings provide a comprehensive multi-omics rationale demonstrating that *GRIN2A* orchestrates neuro-synaptic hijacking, metabolic reprogramming, and immune evasion, thereby validating GluN2 inhibition as a highly viable therapeutic strategy.

The concept that tumors usurp neuronal mechanisms has transitioned from a hypothesis to a defined hallmark [1]. While SCLC has long been recognized for expressing neuroendocrine markers, the functional utility of this lineage has only recently been mapped to active intrinsic electrical activity and the formation of pseudo-synapses with host neurons [11,12,13]. Our functional enrichment analysis aligns perfectly with these discoveries, revealing that *GRIN2A*-high tumors are robustly enriched in neurogenesis, axon development, and synaptic membrane components. As a critical regulatory subunit of the NMDAR complex, GluN2A dictates networks in cancer cells [29]. Upon activation—either by paracrine glutamate calcium permeability and channel gating kinetics [30], which directly couples to oncogenic transcription from innervating nerves or autocrine tumor secretion [31]—calcium influx triggers downstream cascades. Consistently, our GSEA revealed that elevated *GRIN2A* significantly activates oncogenic signaling, including PLCG-ERK, KRAS, and Hedgehog pathways, transforming SCLC cells into proliferative, electrically active networks.

Beyond proliferative signaling, our study uncovered a profound link between *GRIN2A* expression, genomic instability, and metabolic plasticity. High *GRIN2A* expression was correlated with a notably higher mutation frequency of *NOTCH1*, a critical tumor suppressor whose loss drives aggressive SCLC progression [32], and *EP400*, which is a crucial histone acetylation modifier. Our previous studies of genetic profiling have identified that epigenetic regulator *EP300* and *EP400* were frequently mutated in esophageal squamous cell carcinoma, and found a significant association between TMB and *EP300* gene mutations [33,34]. Concurrently, *GRIN2A* overexpression induced a stark metabolic shift, suppressing mitochondrial oxidative phosphorylation, complex I electron transfer, and aerobic respiration. This reprogramming represents a metabolic adaptation: repressing mitochondrial respiration minimizes oxidative stress while potentially redirecting intermediates toward rapid biomass synthesis, simultaneously conferring survival advantages in a hypoxic tumor microenvironment [7,35].

A pivotal translational finding enabled by our immune microenvironment profiling is the profound inverse relationship between *GRIN2A* expression and anti-tumor immunity. *GRIN2A*-high tumors exhibited a distinct “immune-cold” phenotype, characterized by a stark paucity of infiltrating CD8^+^ T cells alongside downregulated antigen-presenting and co-stimulatory molecules (e.g., HLA-B/C, CD40, and CD86). We propose a dual mechanism for this *GRIN2A*-mediated immune exclusion: structurally, the *GRIN2A*-driven suppression of oxidative phosphorylation and the high energy demand of electrical excitability create a nutrient-depleted niche, metabolically restricting effector T cells [7,35,36]; chemically, elevated extracellular glutamate signaling can directly dampen T cell activation and proliferation via immune-expressed glutamate receptors [1,37]. Consequently, *GRIN2A* emerges as a putative biomarker for intrinsic resistance to immune checkpoint blockade in SCLC.

Addressing the therapeutic void in SCLC, our pharmacogenomic analysis revealed that *GRIN2A*-high tumors exhibit distinct vulnerabilities to inhibitors targeting PI3K/mTOR and ERK/MAPK cascades, providing a rational basis for biomarker-guided therapies. Through direct siRNA-mediated perturbation, we provided definitive functional evidence that *GRIN2A* is intrinsically required for SCLC cell proliferation and DNA replication. Most importantly, our experimental validation demonstrated that pharmacological blockade of GluN2 using dizocilpine or memantine potently abrogates SCLC proliferation, migration, 3D spheroid tumorigenicity, and in vivo xenograft growth. Mechanistically, we revealed that memantine actively dismantles essential tumor survival networks—specifically the ERK and AKT signaling cascades—while simultaneously triggering caspase-dependent apoptosis. Memantine, an FDA-approved uncompetitive NMDAR antagonist utilized for Alzheimer’s disease, is particularly promising for immediate clinical translation [23,24,38,39]. It preferentially blocks pathologically over-activated channels while sparing physiological neurotransmission, thus offering an excellent safety profile [40,41]. Given SCLC’s high propensity for brain metastasis—a process strictly reliant on neuronal activity-induced depolarization—memantine’s robust blood–brain barrier permeability [42] offers a unique therapeutic dualism: systematically eradicating primary lesions while simultaneously disrupting the permissive neuro-metastatic niche in the brain. A pharmacological limitation of this study is the requirement for supra-physiological antagonist concentrations in vitro, which is necessary to competitively overcome intense autocrine glutamate loops within a compressed 48 h timeframe. However, our in vivo models successfully suppressed tumor progression using safe, low human-equivalent doses without inducing systemic toxicity. Moving forward, for future clinical translation in human trials, appropriate drug dosages must be rigorously evaluated and optimized to strictly guarantee both safety and therapeutic efficacy.

While our study provides compelling multi-dimensional evidence for *GRIN2A*, limitations exist. Although our in vitro data confirmed marked GRIN2A protein overexpression in SCLC cells relative to normal epithelial cells to corroborate our transcriptomic findings, the lack of an independent clinical cohort currently limits prognostic validation, noting that future studies using larger clinical SCLC cohorts with GRIN2A immunohistochemistry and matched survival data will be important to further validate the protein-level prognostic significance of GRIN2A. Our mechanistic validation primarily relied on cell lines, 3D spheroids, and immunocompromised xenografts. As our current experiments utilized immunodeficient mice, future studies in immunocompetent models are required to validate the in vivo immune functions of *GRIN2A*. It is important to note certain limitations in our study. Furthermore, future studies must employ autochthonous genetically engineered mouse models (GEMMs) to fully dissect the complex neural-immune crosstalk in an immunocompetent setting. Additionally, further patch-clamp electrophysiology is warranted to characterize the precise biophysical properties of *GRIN2A*-mediated currents in SCLC versus normal neurons.

## 5. Conclusions

In summary, this study establishes *GRIN2A* as a crucial node linking pan-cancer neuro-synaptic hijacking, metabolic reprogramming, and immune evasion in SCLC. By integrating large-scale clinical data with rigorous preclinical validations, we reinforce the cancer neuroscience paradigm and propose GluN2-targeted inhibition—specifically via the repurposing of memantine—as an immediately actionable, paradigm-shifting strategy to overcome the lethal recalcitrance of SCLC.

## Data Availability

The datasets presented in this study can be found in online repositories. The names of the repository/repositories and accession number(s) can be found in the article.

## Supplementary Materials

The following supporting information can be downloaded at: https://www.mdpi.com/article/10.3390/biomedicines14061196/s1. Supplementary Figure S1. Prognostic significance of NMDA receptor genes across multiple survival endpoints. Supplementary Figure S2. Prognostic evaluation of NMDAR family genes (except *GRIN2A*) in SCLC. Supplementary Figure S3. Pan-cancer expression landscape and prognostic significance of *GRIN2A*. Supplementary Figure S4. GSEA enrichment analysis between the *GRIN2A*-high and *GRIN2B*-low group using the KEGG dataset. Supplementary Figure S5. Mutational landscape and comparative pathway enrichment of NMDAR family genes in SCLC. Supplementary Figure S6. Validation of GRIN2A knockdown efficiency and cytotoxicity assessment in normal epithelial cells. Supplementary Table S1. Primer sequences used in this research.

## Abbreviations

The following abbreviations are used in this manuscript:

ACC	Adrenocortical Carcinoma
ATCC	American Type Culture Collection
BLCA	Bladder Urothelial Carcinoma
BP	Biological Processes
BRCA	Breast Invasive Carcinoma
CC	Cellular Component
CCK-8	Cell Counting Kit-8
CDX	Cell-Derived Xenograft
CESC	Cervical Squamous Cell Carcinoma and Endocervical Adenocarcinoma
CHOL	Cholangiocarcinoma
COAD	Colon Adenocarcinoma
DFI	Disease-Free Interval
DSS	Disease-Specific Survival
EPIC	Estimating the Proportion of Immune and Cancer cells
ESTIMATE	Estimation of STromal and Immune cells in MAlignant Tumor tissues using Expression data
GDSC	Genomics of Drug Sensitivity in Cancer
GEMMs	Genetically Engineered Mouse Models
GEO	Gene Expression Omnibus
GO	Gene Ontology
GSEA	Gene Set Enrichment Analysis
HNSC	Head and Neck Squamous Cell Carcinoma
HR	Hazard Ratio
IC50	Half-Maximal Inhibitory Concentration
KICH	Kidney Chromophobe
KIRC	Kidney Renal Clear Cell Carcinoma
KIRP	Kidney Renal Papillary Cell Carcinoma
LGG	Brain Lower Grade Glioma
LIHC	Liver Hepatocellular Carcinoma
MF	Molecular Function
NES	Normalized Enrichment Score
NMDA	N-methyl-D-aspartate
NMDAR	N-methyl-D-aspartate Receptor
OS	Overall Survival
OV	Ovarian Serous Cystadenocarcinoma
PAAD	Pancreatic Adenocarcinoma
PBS	Phosphate-Buffered Saline
PCPG	Pheochromocytoma and Paraganglioma
PFI	Progression-Free Interval
PI	Propidium Iodide
PRAD	Prostate Adenocarcinoma
READ	Rectum Adenocarcinoma
SCLC	Small Cell Lung Cancer
SKCM	Skin Cutaneous Melanoma
STAD	Stomach Adenocarcinoma
TCGA	The Cancer Genome Atlas
THCA	Thyroid Carcinoma
THYM	Thymoma
TIMER	Tumor Immune Estimation Resource
TME	Tumor Immune Microenvironment
TPM	Transcripts Per Million
UCEC	Uterine Corpus Endometrial Carcinoma
UCS	Uterine Carcinosarcoma
UVM	Uveal Melanoma
