# Supplementary material for: Pan-Cancer Prognostic Analysis of NMDAR Genes Discovered Therapeutic Implications of Neuronal–Cancer Crosstalk Mediator GRIN2A for Small Cell Lung Cancer
Source: Biomedicines. 2026 May 25;14(6):1196. doi: 10.3390/biomedicines14061196 (PMC13296946; doi:10.3390/biomedicines14061196)
Supplement: Supplementary file 1 [file biomedicines-14-01196-s001.zip › biomedicines-4249722-supplementary.pdf]

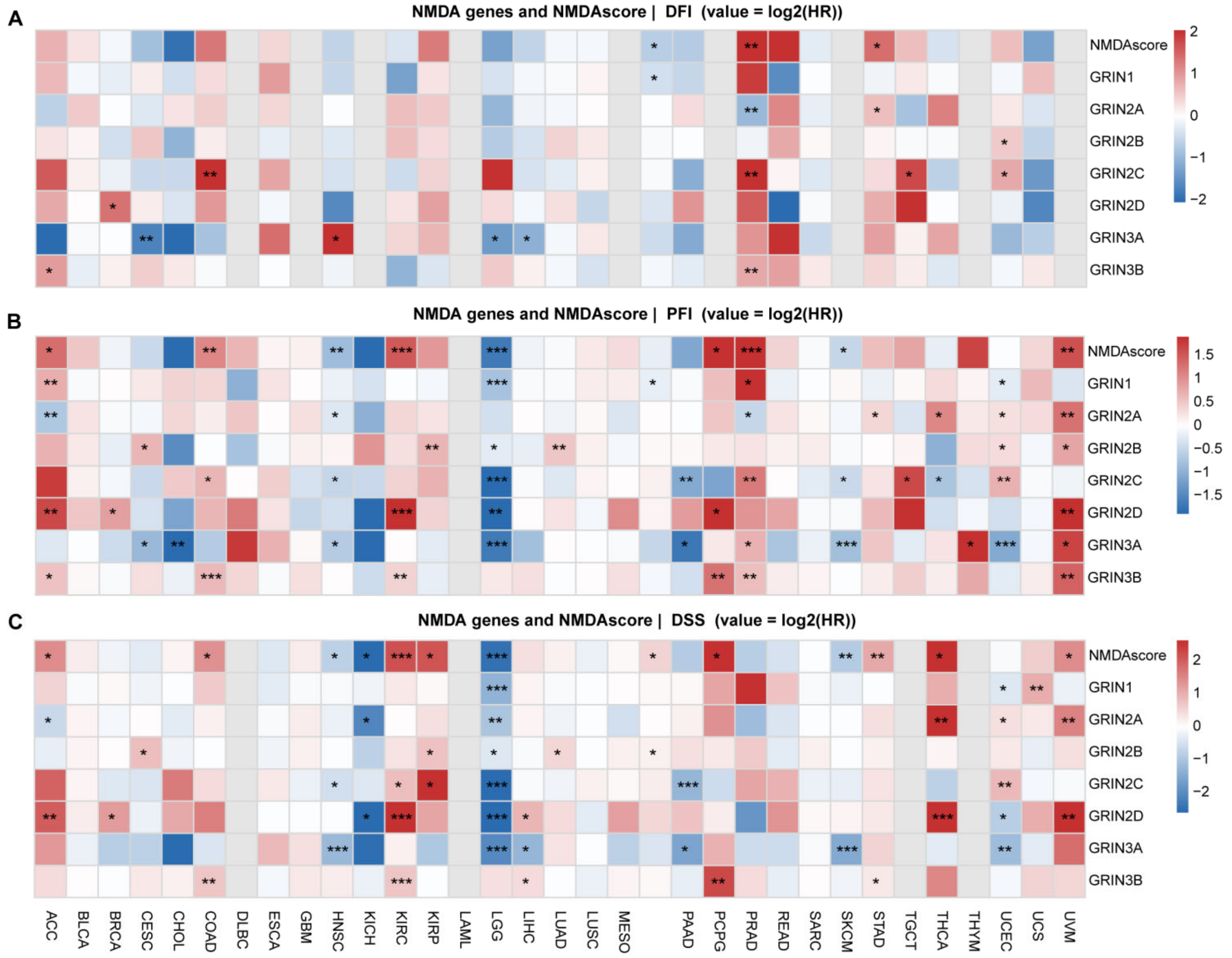

Supplementary Figure S1. Prognostic significance of NMDA receptor genes across multiple survival endpoints. **(A-C)** Heatmaps displaying the results of univariate Cox regression analyses evaluating the association of NMDA receptor genes and the NMDAscore with **(A)** Disease-Free Interval (DFI), **(B)** Progression-Free Interval (PFI), and **(C)** Disease-Specific Survival (DSS) across pan-cancer cohorts. The color gradients represent the log2(Hazard Ratio) values. Statistical significance was determined using the log-rank test. *p < 0.05, **p < 0.01, ***p < 0.001.

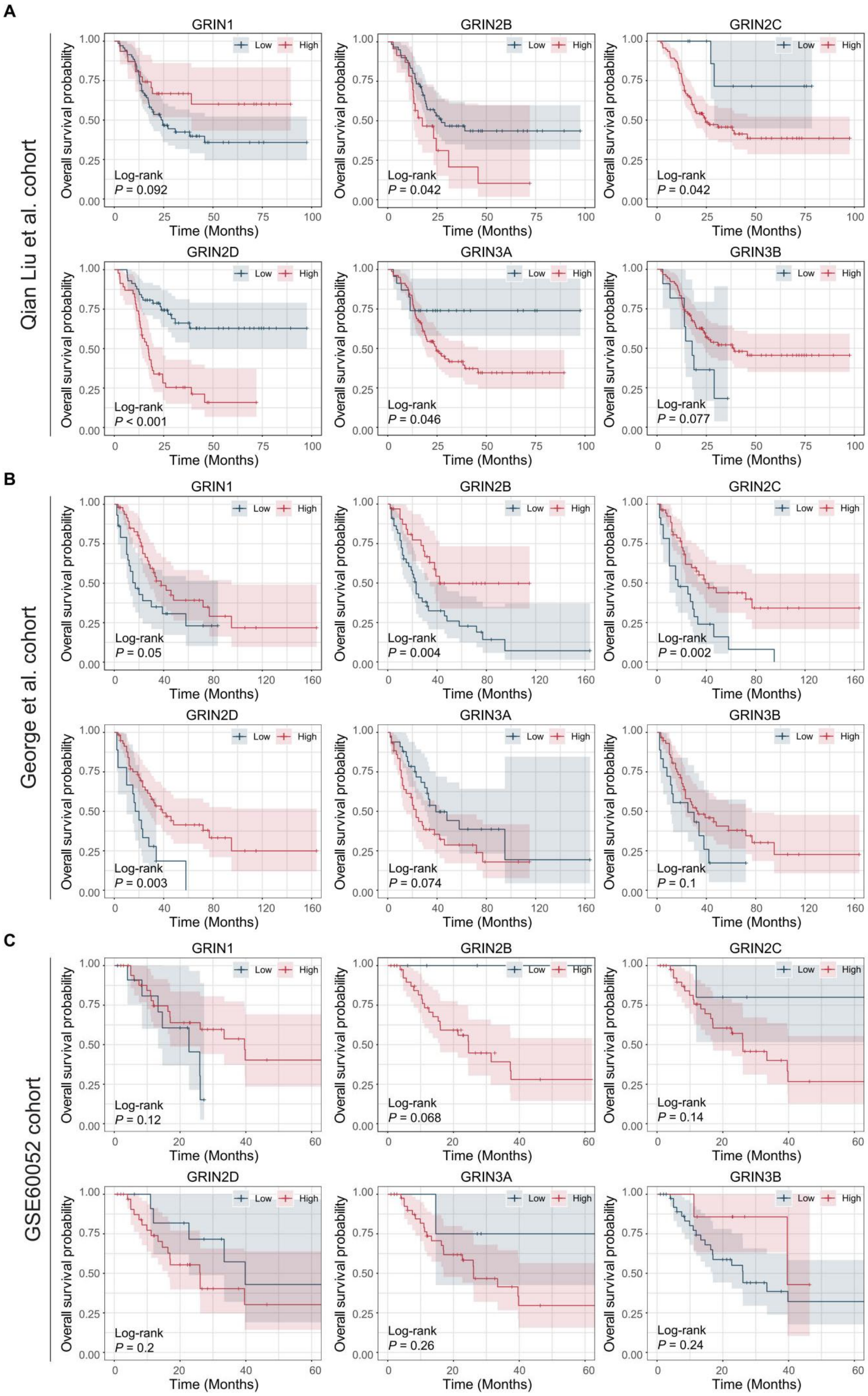

Supplementary Figure S2. Prognostic evaluation of NMDAR family genes (except *GRIN2A*) in SCLC. Kaplan-Meier survival curves of OS for SCLC patients stratified into high and low

expression groups based on the median expression levels of *GRIN1*, *GRIN2B*, *GRIN2C*, *GRIN2D*, *GRIN3A*, and *GRIN3B* in the Qian Liu et al.cohort **(A)**, George et al. cohort **(B)**, and the GSE60052 cohort **(C)**. The log-rank test was utilized to calculate p-values.

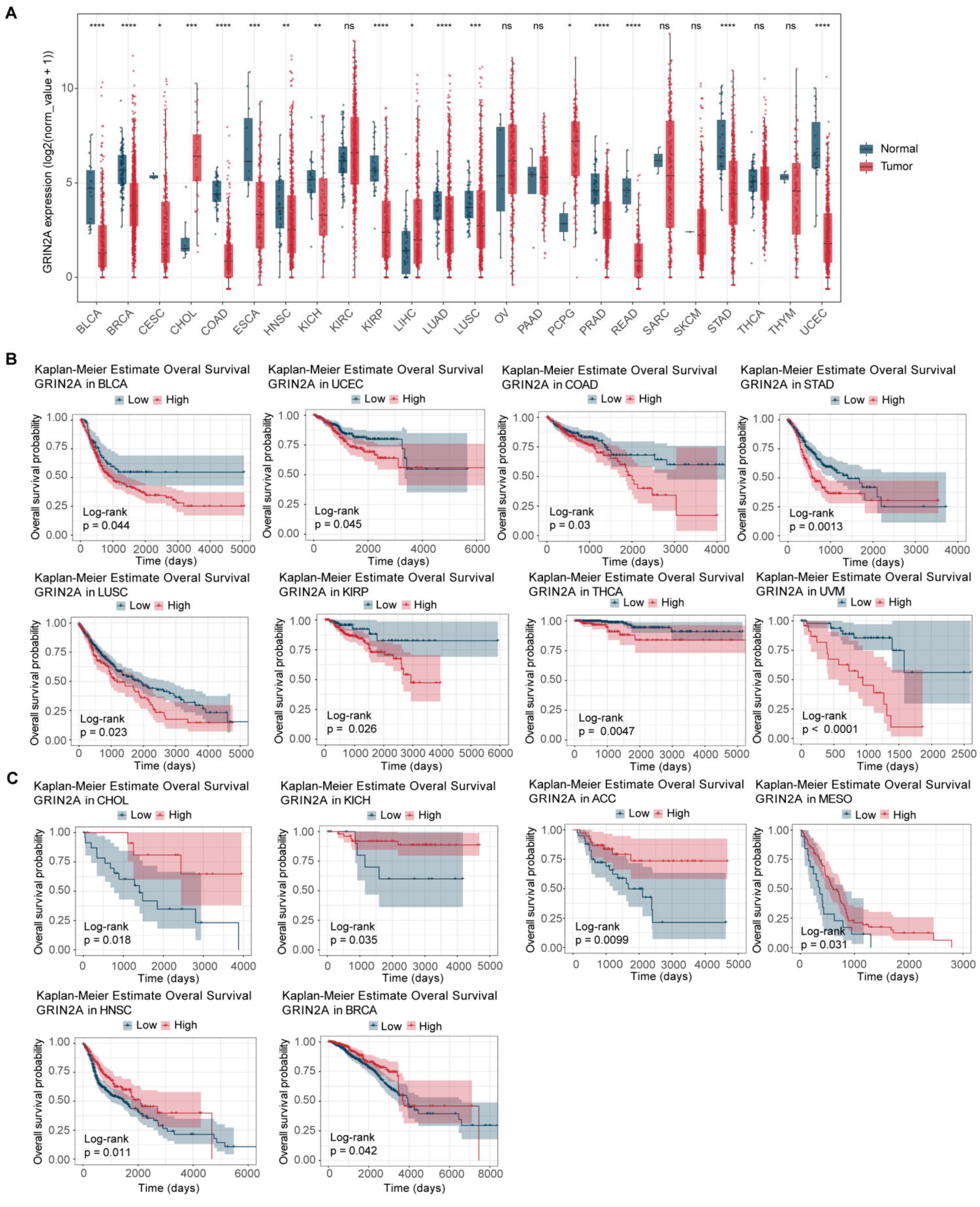

Supplementary Figure S3. Pan-cancer expression landscape and prognostic significance of *GRIN2A*. **(A)** Boxplot illustrating the differential mRNA expression of *GRIN2A* between tumor tissues and corresponding normal tissues across the TCGA pan-cancer cohorts. **(B-C)** Kaplan-Meier survival curves of OS for patients stratified by high and low *GRIN2A* expression across various

indicated cancer types (BLCA, UCEC, COAD, STAD, LUSC, KIRP, THCA, UVM, CHOL, KICH, ACC, MESO, HNSC, and BRCA). Statistical comparisons were performed using the log-rank test. $^*p < 0.05$, $^{**}p < 0.01$, $^{***}p < 0.001$, $^{****}p < 0.0001$.

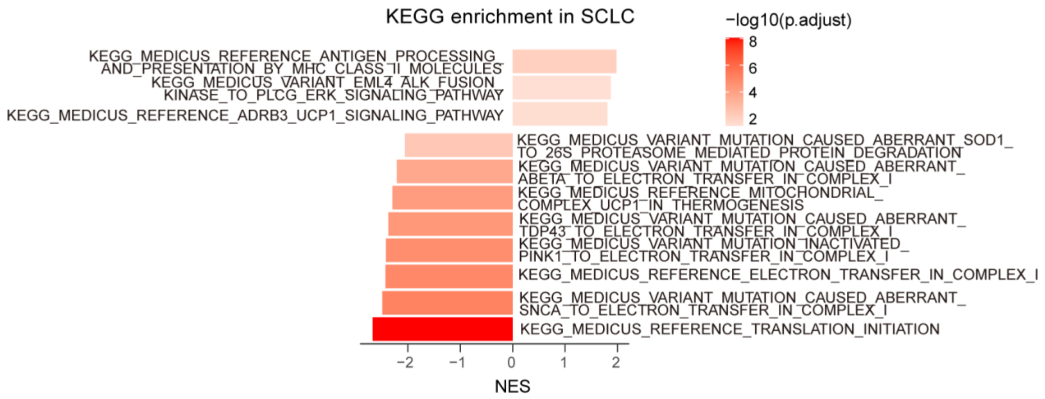

Supplementary Figure S4. GSEA enrichment analysis between the *GRIN2A*-high and *GRIN2B*-low group using the KEGG dataset.

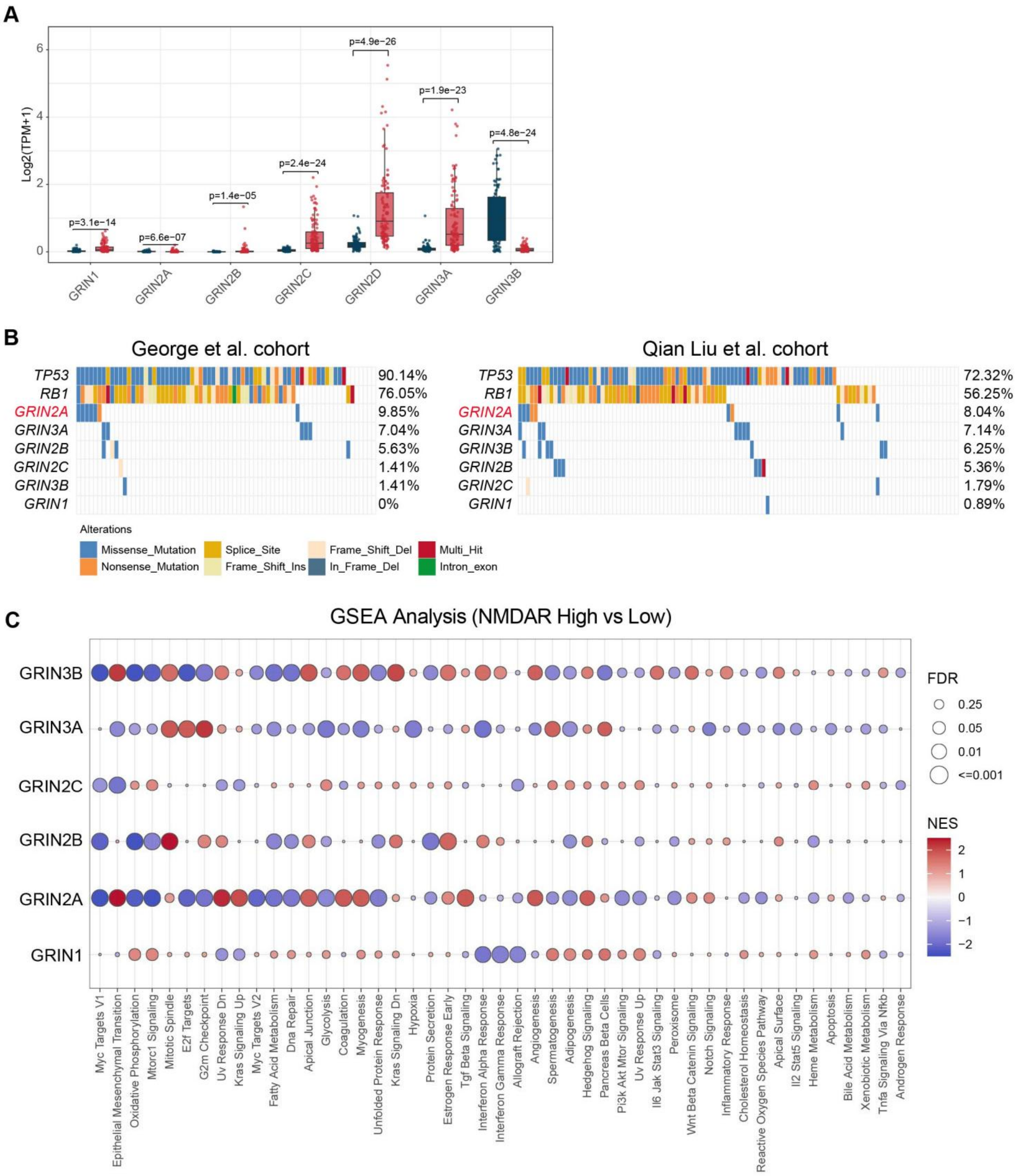

Supplementary Figure S5. Mutational landscape and comparative pathway enrichment of NMDAR family genes in SCLC. **(A)** Boxplots displaying the mRNA expression levels of NMDAR family genes (GRIN1, GRIN2A-D, GRIN3A-B) in SCLC tumor tissues compared to adjacent normal tissues. **(B)** Oncoplots displaying the somatic mutation frequencies of TP53, RB1, and major NMDAR genes in the George et al. cohort and the Qian Liu et al. cohort. **(C)** Bubble plot showing comparative GSEA of Hallmark pathways between high and low expression groups across individual

NMDAR subunits. Statistical comparisons were performed using the log-rank test. *$p < 0.05$, **$p < 0.01$, ***$p < 0.001$, ****$p < 0.0001$.

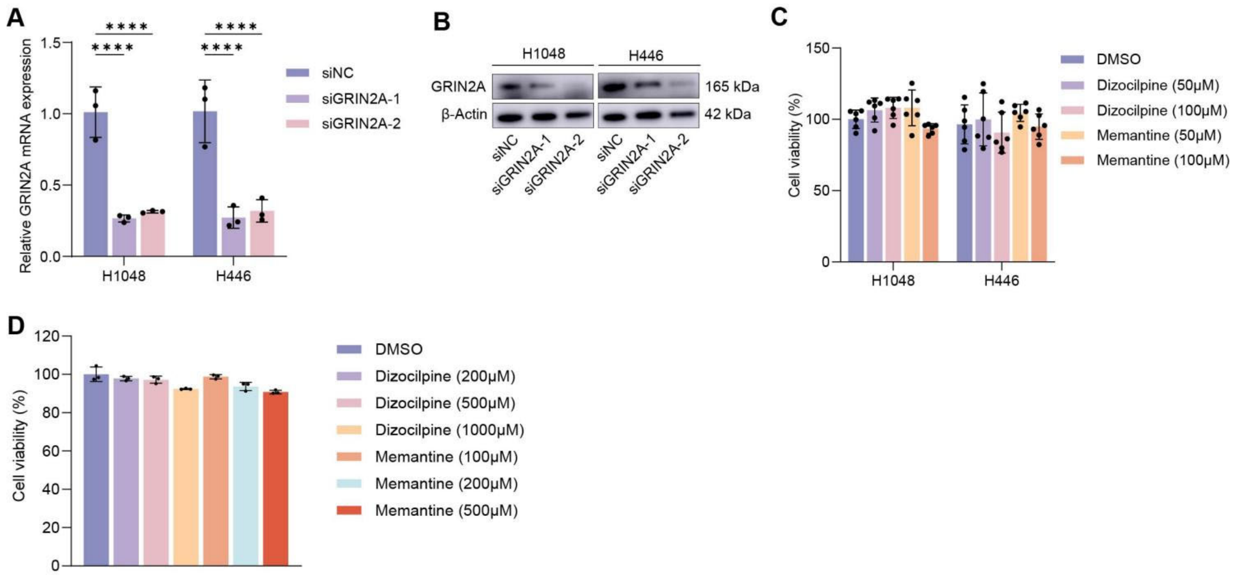

Supplementary Figure S6. Validation of GRIN2A knockdown efficiency and cytotoxicity assessment in normal epithelial cells. **(A)** RT-qPCR and Western Blot **(B)** analysis evaluating the knockdown efficiency of GRIN2A in H1048 and H446 cells following GRIN2A knockdown. **(C)** Cell viability of H1048 and H446 cells treated with DMSO or low doses (50 μM and 100 μM) of dizocilpine or memantine for 48h, evaluated by CCK-8 assay. **(D)** Cell viability of the BEAS-2B treated with indicated concentrations of dizocilpine or memantine for 48h, assessed by CCK-8 assay. Data are presented as mean ± SD. ****$p < 0.0001$.

Supplementary table 1. Primer sequences used in this research

Gene	Species	Forward primer (5'->3')	Forward primer (5'->3')
GRIN2A	Human	GACCCCAAGAGCCTCATCAC	CTGGATGGACGCTCCAAACT
ACTB	Human	AGGATTCCTATGTGGGCGAC	ATAGCACAGCCTGGATAGCAA
siGRIN2A-1	Human	GCAACAUCUGGAAGAACUATT	UAGUUCUUCCAGAUGUUGCTT
siGRIN2A-2	Human	GCUUCAUCAUCCAGCACUATT	UAGUGCUGGAUGAUGAAGCTT
